# Non-pharmacological pain treatments as neuro-epigenetic revalidation strategies: integrating chronobiology, acupuncture, and pharmaco-nutrition

**DOI:** 10.3389/fpain.2026.1786367

**Published:** 2026-04-23

**Authors:** Agnès Mazic de Sonis, Corinne Granger

**Affiliations:** 1 Pain Clinic Pole Neuroscience, Chirec Delta Hospital, Brussel, Belgium; 2 Stella Polaris Europe, Paris, France

**Keywords:** chronobiology, epigenetics, integrative pain medicine, non-pharmacological pain treatments, nutrition, P4medicine personalized medicine

## Abstract

**Background:**

Chronic pain is increasingly conceptualized as a disorder of maladaptive neural plasticity sustained by central sensitization, neuroinflammation signalling, disrupted biological rhythms, metabolic dysregulation and environmentally mediated epigenetic modulation, and altered gut–brain interactions. While pharmacological approaches remain central to pain management, their long-term efficacy is limited by tolerance, adverse effects, opioid-induced hyperalgesia, and inter-individual variability. Growing evidence suggests that non-pharmacological interventions may modulate pain not only symptomatically, but through deeper neurobiological and epigenetic mechanisms.

**Hypothesis:**

We propose that selected non-pharmacological pain treatments, particularly chronobiology-informed acupuncture and targeted pharmaco-nutrition, may be considered as neuro-epigenetic revalidation strategies. These interventions may restore adaptive gene expression profiles and neural, immune, metabolic and circadian regulation disrupted in chronic pain, thereby reducing nociceptive sensitization and improving treatment responsiveness.

**Rationale:**

Acupuncture has been shown to modulate central pain networks, including prefrontal, limbic, and sensorimotor regions, with effects distinct from sham procedures. Chronobiological regulation of sleep–wake cycles, hormonal rhythms, and feeding timing influences inflammatory pathways and epigenetic regulation. In parallel, the gut–brain axis, through intestinal barrier integrity, microbiome composition, and immune–glial signalling, plays a critical role in pain chronification and drug metabolism. Nutritional and nutraceutical interventions can influence these pathways and have been associated with changes in inflammatory tone, opioid tolerance, and neuroimmune interactions.

**Testable predictions:**

This framework generates testable predictions linking multimodal interventions to dynamic modulation of epigenetic signatures (DNA methylation, microRNA expression), chronobiological and sleep parameters, inflammatory mediators, gut-brain markers and clinically meaningful outcomes. Longitudinal, multimodal study designs are required to evaluate association between regulatory recalibration and sustained clinical improvement.

**Conclusion:**

Viewing non-pharmacological pain treatments within a neuro-epigenetic revalidation model provides a coherent system-level perspective model that bridges neuroscience, chronobiology, epigenetics, and integrative pain medicine. This integrative model supports the development of personalized, mechanism-based strategies for chronic pain management while encouraging biomarker-informed translational research.

## 1 Introduction

Chronic pain represents a major clinical and societal burden, affecting a substantial proportion of the global population and frequently persisting despite advances in pharmacological management. Over the past decades, progress in neuroscience has profoundly transformed the understanding of pain, shifting it from a purely nociceptive phenomenon to a complex disorder of neural plasticity. Chronic pain is now widely conceptualized as a state of maladaptive sensitization involving peripheral nociceptors, spinal processing, supraspinal networks, and neuroimmune interactions (1).

Despite this conceptual evolution, treatment outcomes remain highly variable. Long-term pharmacological strategies, particularly those relying on opioids and adjuvant analgesics, are limited by tolerance, adverse effects, drug–drug interactions, and inter-individual variability in response. A key clinical expression of sensitization is hyperalgesia, including medication-driven forms such as opioid-induced hyperalgesia, which may contribute to treatment failure and chronicity (2–4). These limitations highlight the need for complementary approaches capable of addressing the biological processes underlying pain chronification rather than solely suppressing symptoms.

Converging evidence indicates that chronic pain is associated with persistent alterations in gene expression, sustained neuroinflammation signalling, glial activation, and altered connectivity within pain-related brain networks (5). These processes are influenced by environmental and behavioural factors such as stress, sleep disruption, circadian misalignment, nutrition, and pharmacological exposure. Epigenetic mechanisms, including DNA methylation, histone modifications, and microRNA regulation, provide a biological interface through which such exposures can induce stable yet potentially reversible changes in neural and immune function.

In parallel, non-pharmacological interventions such as acupuncture (6) chronobiology-based strategies, and targeted nutritional approaches have demonstrated clinically meaningful effects in chronic pain conditions. Acupuncture has been shown to modulate central pain networks (7), activate descending inhibitory pathways involved in endogenous opioid analgesia and regulated autonomic balance on acting on vagal pathways, enhancing parasympathetic tone while reducing sympathetic activity (8, 9).

Chronobiological regulation of sleep–wake cycles and feeding rhythms influences inflammatory tone, stress responsiveness and neuro endocrine regulation. Nutrition and nutraceuticals further interact with pain pathways through modulation of the gut–brain axis, intestinal barrier integrity, microbiome composition, and drug metabolism.

These approaches are frequently studied in isolation and interpreted primarily through symptomatic endpoints. However, their effects share convergent biological substrates. We propose that selected non-pharmacological pain treatments can be conceptualized within a neuro-epigenetic framework, in which multimodal interventions contribute to restoring maladaptive plasticity that has become dysregulated in chronic pain.

In this article, we advance the hypothesis that chronobiology-informed acupuncture and pharmaco-nutrition strategies may operate as neuro-epigenetic revalidation mechanisms.

The term *revalidation* is used to describe a process of restoring adaptive regulatory flexibility within dysregulated biological and neurobiological systems. Rather, it refers to progressive recalibration of interacting neural, immune, metabolic, and circadian networks toward functional coherence.

This framework aims to bridge neuroscience, epigenetics, and integrative pain medicine, while generating testable predictions for future longitudinal and translational research.

## 2 Neuro-Epigenetic revalidation: conceptual model

### 2.1 From maladaptive plasticity to revalidation

Chronic pain is increasingly recognized as a condition characterized by persistent maladaptive plasticity. Repeated nociceptive input, sustained neuroinflammation, and biological and psychological stressors, as well as chronic pharmacological exposure, may contribute to long-lasting alterations in neural circuits involved in sensory processing and affective modulation of pain perception.

These changes are accompanied by modifications in gene expression profiles within neurons, glial and immune cells, increasing sensitization and functional rigidity.

Epigenetic regulation provides a plausible mechanistic substrate for the persistence of these alterations. By modulating chromatin accessibility and transcriptional activity without altering the DNA sequence, epigenetic mechanisms enable environmental and behavioural influences to become biologically embedded. In chronic pain, such mechanisms may stabilize pro-inflammatory signalling, enhance excitatory neurotransmission, impair inhibitory control, and consolidate pain-related memory traces.

Within this context, the concept of neuro-epigenetic revalidation is proposed as a systemic level adaptative process. Unlike the notion of “epigenetic resetting,” which often implies targeted reversal of specific molecular pathways, neuro-epigenetic revalidation refers to coordinated regulatory recalibration across interacting biological systems. These include neural networks, immune pathways, metabolic regulation, and circadian organization.

Rather than focusing on isolated molecular targets, revalidation aims to restore regulatory coherence between systems whose dysregulation contributes to pain chronification. Hyperalgesia is interpreted within this framework as a clinical multimodal systemic mechanism, maintained by neuroimmune activation (1), altered synaptic plasticity, and epigenetic stabilized patterns.

### 2.2 Non-pharmacological interventions as modulators of regulatory plasticity

Non-pharmacological pain treatments share a common feature: they engage endogenous regulatory systems rather than introducing exogenous pharmacological pressure.

#### 2.2.1 Acupuncture and patterned sensory input

Acupuncture remains an area of ongoing scientific debate, with variability in clinical outcomes reflecting heterogeneity in stimulation parameters, treatment timing, and patient stratification (9). Mechanistic investigations nevertheless indicate engagement of limbic, prefrontal, and descending inhibitory networks, including activation of endogenous opioid and adenosine pathways (10, 11). Neuroimaging studies consistently report modulation of functional connectivity within pain-related networks.

Comparisons with diffuse noxious inhibitory control (DNIC) paradigms suggest that patterned somatosensory input may activate overlapping descending modulatory systems. In addition, placebo analgesia—now recognized as a biologically mediated phenomenon—operates through expectation-driven engagement of endogenous inhibitory circuits rather than purely psychological mechanisms (9).

Beyond immediate network modulation, transdermal electrical stimulations (12, 13) such as TENS and electroacupuncture have demonstrated alterations in spinal neurotransmitter dynamics, including reductions in excitatory glutamatergic transmission and modulation of endogenous opioid pathway. Sustained changes in neurotransmission may influence intracellular calcium-dependent pathways, kinase activation cascades, and activity-dependent transcriptional programs.

Because neuronal activity-dependent transcription is closely coupled to chromatin remodelling processes, repeated patterned neuromodulator input may plausibly contribute to longer-term regulatory recalibration. In parallel, autonomic modulation through vagal pathways may attenuate systemic inflammatory tone, indirectly shaping immune and glial activation states.

Within the neuro-epigenetic revalidation framework, acupuncture is therefore conceptualized not as an isolated symptomatic technique, but as a structured neuromodulator input capable of engaging endogenous plasticity mechanisms across neural and autonomic systems. While direct epigenetic effects remain to be demonstrated in chronic pain populations, these convergent neurochemical and autonomic pathways provide biologically plausible links between patterned sensory stimulation and systemic regulatory modulation.

#### 2.2.2 Chronobiological regulation

Temporal disorganization represents another dimension of chronic pain dysregulation. Disrupted circadian rhythms influence immune function, hormonal secretion, sleep architecture, and gene expression dynamics (14, 15). Many epigenetic processes exhibit circadian oscillations.

Chronobiological alignment through sleep–wake synchronization, timed light exposure, structured daily routines, and temporal coordination of interventions may therefore support restoration of regulatory coherence. Chrono-acupuncture and timing of pharmacological administration represent potential translational extensions of this principle.

Rather than acting in isolation, chronobiological strategies may enhance the efficacy of other interventions by stabilizing the temporal context and thereby potentially allowing synergistic effects.

#### 2.2.3 Pharmaco-nutrition and the gut-brain axis

Nutrition and nutraceutical strategies contribute to regulatory modulation primarily through their interaction with the gut–brain axis (16). The intestinal microbiome plays a central role in immune regulation, neuroactive metabolite production, and drug metabolism. Dysbiosis and impaired intestinal barrier function have been associated with systemic inflammation, altered nociceptive signalling, opioid tolerance, and variability in pharmacological response during pain chronification (3, 4, 17).

Beyond micronutrient sufficiency, macronutrient composition itself may critically influence epigenetic regulation. Lipid signalling pathways operate upstream of chromatin remodelling mechanisms. Fatty acid-derived mediators and membrane lipid composition modulate inflammatory cascades, nuclear receptor activation (e.g., PPARs), and intracellular signalling pathways that regulate histone acetylation and DNA methylation. In parallel, key metabolic intermediates such as acetyl-CoA, S-adenosylmethionine (SAM), and NAD^+^ serve as substrates or cofactors for epigenetic enzymes, directly linking nutritional state to chromatin accessibility and transcriptional activity. Dietary patterns characterized by altered lipid quality, excessive refined carbohydrates, or protein imbalance may therefore shape the epigenetic landscape through metabolic and inflammatory pathway.

Targeted nutritional rehabilitation may thus support regulatory recalibration by modifying immune tone, mitochondrial function, and metabolic inputs to the nervous system (16, 18). Adequate intake of key micronutrients such as magnesium and selenium remains essential for mitochondrial metabolism (19), redox balance, and DNA repair processes, and correction of subclinical deficiencies may be particularly relevant in patients exposed to long-term polypharmacy.

In addition, several phytonutrients and candidate senolytic compounds—including resveratrol (16), quercetin, curcumin (20), fisetin, and hydroxytyrosol—have been investigated for their capacity to modulate oxidative stress signalling and attenuate senescence-associated pro-inflammatory phenotypes.

Lifestyle-related metabolic modulators may further amplify these effects. Individualized physical exercise can induce adaptive stress responses (mitohormesis), supporting mitochondrial biogenesis and metabolic flexibility.

Fasting and caloric restriction paradigms have been associated with activation of autophagy-related and cellular repair pathways. NAD^+^ precursors, such as nicotinamide riboside, warrant further investigation given their potential influence on sirtuin signalling, mitochondrial turnover, and genomic stability.

Collectively, these mechanisms position nutritional and metabolic regulation as upstream drivers capable of influencing immune activity, mitochondrial resilience, and epigenetic plasticity within the neuro-epigenetic revalidation model.

Light-based interventions represent another emerging non-pharmacological approach with potential relevance for chronic pain revalidation. Transcranial (21) or targeted photobiomodulation therapies (22) may exert analgesic and anti-inflammatory effects. Proposed mechanisms include retinal photoreceptor signalling, direct mitochondrial regulation, and downstream modulation of neural network activity.

Future work should prioritize parameter standardization (wavelength, dose, timing) (21–24), phenotypic stratification (hyperalgesia/sensitization profiles), and integration of objective biomarkers and clinical outcomes.

### 2.3 Toward an integrated hypothesis

We propose that the clinical effects observed with multimodal non-pharmacological pain management (10) can be interpreted as manifestations of neuro-epigenetic revalidation. In this integrative model, acupuncture, chronobiology regulation and pharmaco-nutrition converge on shared biological cellular targets including neuroinflammation, glial activation, network connectivity, and gene expression dynamics (10).

**Figure 1:**
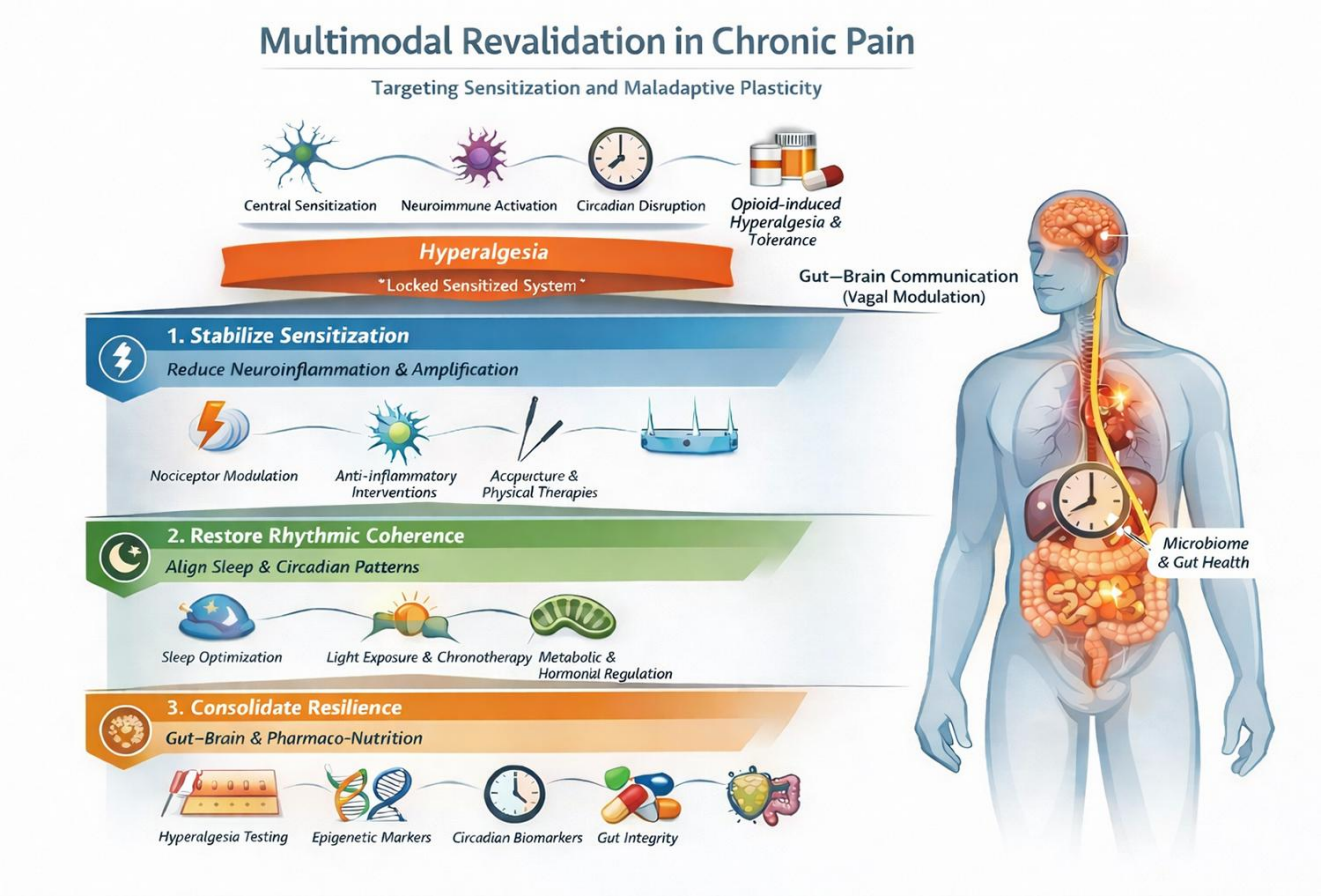
Conceptual model of neuro-epigenetic revalidation in chronic pain. The model illustrates chronic pain as a state of systemic dysregulation and outlines a three-tier strategy aimed at reducing sensitization and restoring regulatory balance.

By reducing pathological amplification and restoring temporal and metabolic coherence, these interventions may promote a transition from a rigid sensitized, pain state toward a more flexible and resilient regulatory phenotype (Figure 1).

This transition does not imply uniform effects across individuals; inter-individual variability in genetic background, epigenetic susceptibility, microbiome composition, and life history will shape treatment responsiveness (16, 25). Importantly, this variability also offers an opportunity for stratification and personalization.

Framing non-pharmacological interventions within this conceptual scaffold provides a theoretical basis for integrating clinical practice with molecular and systems-level research.

It invites identification of biomarkers panels, capable of capturing treatment-induced modulation, and supports the design of longitudinal, multimodal studies to test causality and durability of effects.

## 3 Testable predictions and biomarkers

A central strength of the neuro-epigenetic revalidation hypothesis lies in its capacity to generate testable predictions across molecular, physiological, and clinical domains. If multimodal non-pharmacological interventions contribute to restoring adaptive regulatory states, their effects should be detectable through convergent biomarkers reflecting modulation of gene expression, neuroimmune signalling, chronobiological rhythms, and pain-related network function outcomes (Table 1).

**Table 1:** Neuro-epigenetic revalidation framework: testable predictions, biomarkers, and outcomes.

Domain	Testable prediction	Candidate biomarkers	Assessment methods	Expected clinical outcomes
Epigenetic regulation	Non-pharmacological interventions induce reversible modulation of pain-related gene expression	DNA methylation patterns (inflammatory, stress-response, opioid-related genes); microRNA profiles	Peripheral blood epigenetic assays (longitudinal)	Reduced pain persistence, improved treatment responsiveness
Neuroimmune signaling	Reduction of low-grade neuroinflammation associated with chronic pain	Pro- and anti-inflammatory cytokines; immune-glial interaction markers	Blood-based inflammatory panels	Decreased pain intensity and variability; reduced central sensitization
Neural plasticity	Restoration of adaptive pain network dynamics	Indirect functional markers of central sensitization; network-level proxies	Functional imaging (when available); validated central sensitization scales	Improved pain modulation; reduced affective burden of pain
Chronobiology	Re-alignment of disrupted biological rhythms	Sleep duration and efficiency; circadian rhythm stability; cortisol profiles	Actigraphy; sleep questionnaires; salivary cortisol	Improved sleep quality, fatigue reduction, enhanced resilience
Gut–brain axis	Improved intestinal barrier and microbiome-related modulation of pain	Markers of gut permeability; microbiome diversity/composition; metabolic markers	Stool analysis; blood markers; clinical GI assessments	Reduced inflammation, improved drug tolerance and efficacy
Pharmacological interaction	Decreased need for high-dose analgesics	Analgesic consumption (opioids, adjuvants); side-effect profiles	Medication tracking; clinical follow-up	Dose reduction, fewer adverse effects, improved quality of life
Global clinical outcome	Transition from rigid sensitized state to flexible adaptive regulation	Composite clinical scores (pain, QoL, sleep, fatigue)	Multidimensional patient-reported outcome measures	Sustained functional improvement beyond symptom suppression

For each domain (epigenetic regulation, neuroimmune signalling, neural plasticity, chronobiology, gut–brain axis, pharmacological interaction, and global clinical outcomes), the table outlines (i) the conceptual testable prediction, (ii) candidate biological markers, (iii) potential assessment methods, and (iv) expected clinical correlates. The table is intended to operationalize the hypothesis and guide longitudinal multimodal research designs rather than to imply established causal relationships.

Rather than assuming direct causality, this framework proposes measurable associations between intervention-induced regulatory changes and clinically meaningful improvement. Longitudinal assessment is therefore essential to distinguish transient compensatory responses from sustained recalibration.

### 3.1 Epigenetic signatures

One primary prediction of this framework is that effective multimodal interventions will be associated with dynamic modulation of epigenetic markers implicated in pain processing and inflammation. Candidate biomarkers may include DNA methylation patterns of genes, implicated in cytokine regulation, inflammatory signalling cascade opioid receptors pathways, and stress-response systems, as well as with microRNA expression profiles associated with neuroinflammation, neuroimmune crosstalk and glial activation.

Importantly, the revalidation model anticipates partial reversibility and dynamic fluctuation rather than permanent reprogramming. Such changes, if correlated with clinical improvement, would support the concept of adaptive regulatory recalibration.

### 3.2 Neuroimmune and inflammatory biomarkers

Given the central role of low-grade neuroinflammation and glial activation in chronic pain, another testable prediction is normalization or attenuation of inflammatory signalling following effective intervention. Circulating pro- and anti-inflammatory cytokines (e.g.,IL-6, TNF-α, IL-1β)), chemokines involved in immune glial crosstalk such as (CCL2/MCP-1), emerging glial-related biomarkers including GFAP and S100B, as well as regulatory mediators such as sirtuins (particularly SIRT1 and SIRT3), may provide accessible peripheral proxies of central neuroimmune tone and central sensitization dynamics (5, 25–27).

While peripheral markers cannot fully capture central processes, convergent reductions in inflammatory mediators accompanied by improved pain modulation would strengthen the plausibility of systemic regulatory effects. These associations should be interpreted cautiously, as inflammatory shifts may represent downstream consequences rather than primary drivers of improvement (1).

### 3.3 Chronobiological and physiological markers

As chronobiological regulation plays a key role in the proposed model, effective revalidation is expected to translate into improved sleep quality, reduced fatigue, and better alignment of daily biological rhythms. These outcomes may be objectively evaluated using actigraphy-derived sleep metrics, circadian rhythm stability indices, normalization of diurnal cortisol profiles, and chronotype assessments (28, 29).

Improved alignment between subjective fatigue, stress management (29), sleep quality, and pain intensity would further support the hypothesis that regulatory flexibility, rather than isolated symptom suppression, is being restored. Such measures provide non-invasive markers interpretated into pragmatic clinical designs.

### 3.4 Gut-brain axis indicators

Interventions targeting nutrition and intestinal function are predicted to influence pain outcomes through modulation of gut–brain signalling (16). Biomarkers of intestinal barrier integrity, systemic endotoxemia, microbiome diversity and composition, and metabolic signatures may provide indirect evidence of improved regulatory balance (30).

In patients receiving analgesic pharmacotherapy, modulation of drug metabolism markers or reduction in dose escalation may reflect improved systemic resilience. Correlations between gut-related biomarkers, inflammatory shift, and clinical improvement would support the integrative nature of the model.

### 3.5 Clinical and functional outcomes

At the clinical level, neuro-epigenetic revalidation should manifest as more than transient analgesia. Predicted outcomes include sustained reductions in pain intensity and variability, improvements in sleep quality, and fatigue, reduced reliance on analgesic dose escalation-particularly opioids- and enhanced overall functional capacity.

Improved treatment responsiveness over time suggests increased system flexibility rather than simple suppression of pain signals (31–33).

The synergistic multimodal approach is anticipated to translate into improved responsiveness to therapeutic interventions and attenuation of nociceptive signal amplification. Multidimensional patient-reported outcome measures are therefore essential to capture changes extending beyond pain intensity alone (34).

### 3.6 Implications for study design

Evaluating multimodal regulatory strategies presents methodological challenges, as traditional randomized controlled trial designs may inadequately capture system-level dynamics, and interdependent mechanisms. Longitudinal, multimodal and “whole-system” study designs integrating clinical endpoints with biological sampling and chronobiological monitoring are better suited to testing the revalidation hypothesis.

Pain measurement is inherently subjective, with self-report remaining the gold standard, as no fully objective measurement method currently exists.

The efficacy (performance in ideal conditions) and effectiveness (real-world performance) of pain measurement tools are generally high for acute, postoperative, and chronic pain, provided they are chosen based on the patient's population and cognitive status.

Such approaches are better suited to evaluate complex interventions, rather than isolating single variables, and may help distinguish revalidation processes from placebo effects or short-term compensatory changes.

Importantly, epigenetic associations should be interpreted as they do not imply direct causality and observed modifications may reflect downstream consequences rather than primary drivers of clinical improvement. Strengthening causal inference will require careful study design, repeated measures, and triangulation across biological domains. Finally, the proposed framework predicts heterogeneity of response; stratifying participants according to baseline epigenetic, chronobiological, or microbiome profiles may help identify responders and non-responders and support the development of personalized, mechanism-based pain management strategies (35).

The neuro-epigenetic revalidation hypothesis becomes empirically approachable while preserving appropriate scientific caution.

## 4 Discussion and clinical implications

This article proposes that non-pharmacological pain treatments may be conceptualized as biologically active modulators of maladaptive plasticity operating through neuro-epigenetic mechanisms. By integrating acupuncture, chronobiological regulation, and pharmaco-nutrition, the neuro-epigenetic revalidation hypothesis aligns these interventions as potential synergistic contributors of system-level organization in chronic pain states rather than merely adjunctive therapies (11, 22, 30, 31).

This perspective aligns with contemporary neuroscience, which increasingly conceptualizes chronic pain as a dynamic condition maintained by altered network connectivity, persistent neuroinflammation, dysregulation modulating gene expression (36). Epigenetic regulation offers a plausible substrate linking environmental and behavioural inputs to durable neurobiological changes, while remaining closed to established pharmacological treatments.

Importantly, metabolic and nutritional signalling pathways provide mechanistic continuity between environmental exposures and epigenetic regulation. Lipid-derived mediators, mitochondrial metabolism, and NAD^+^-dependent sirtuin activity represent convergent nodes through which diet and energy balance may influence transcriptional stability in neural and immune cells.

From a clinical standpoint, adopting a neuro-epigenetic revalidation framework shifts therapeutic objectives from short-term symptom suppression toward restoration of adaptive regulation. Improvements are therefore expected to extend beyond pain intensity, sleep quality, fatigue, emotional regulation, medication burden, and overall resilience. This systems-level orientation may also support earlier integration of non-pharmacological interventions to mitigate chronification, reduce the need for dose escalation, and limit iatrogenic complications. Importantly, it provides a rationale for personalization, as inter-individual variability (genetic, epigenetic, microbiome-related, and psychosocial) is likely to influence responsiveness and may enable stratified treatment approaches.

A key therapeutic implication is the need to address hyperalgesia, understood as an amplified pain response driven by altered cellular and nociceptor excitability, spinal facilitation, and supraspinal network dysregulation. Hyperalgesia can emerge not only because of tissue injury and inflammation but also as a self-sustaining state maintained by neuroimmune signalling, circadian disruption, and maladaptive plasticity. Hyperalgesia represents a clinically observable marker of a “locked” sensitized system and should guide therapeutic sequencing and outcome assessment.

At the mechanistic level, sensitization reflects interactions between ion-channel regulation, intracellular signalling, neuroglial activation, and microenvironmental factors, ultimately producing a persistent hyperexcitable nociceptive state.

Moreover, chronic pharmacological pressure may generate or exacerbate sensitization, and opioid-induced hyperalgesia (OIH), together with tolerance and adverse effects, represents a major limitation to long-term analgesic efficacy (4).

Clinically, the neuro-epigenetic revalidation hypothesis supports a strategic shift: rather than escalating analgesic regimens in a sensitized system, treatment should prioritize recovery of regulatory flexibility through coordinated multimodal interventions targeting nociceptive amplification, neuroimmune tone, circadian integrity, and gut–brain interactions.

A pragmatic strategy can be conceptualized as a three-layer multimodal approach:

(i) stabilizing the hyperalgesia state by reducing amplification and neuroinflammatory drive (acupuncture as a patterned sensory neuromodulator input (8, 9, 25, 31, 32),
(ii) restoring circadian and metabolic coherence through chronobiological revalidation, and
(iii) consolidating resilience through gut–brain and pharmaco-nutritional optimization, with attention to medication-related vicious circles (e.g., constipation, dysbiosis, micronutrient depletion).

**Figure 2:**
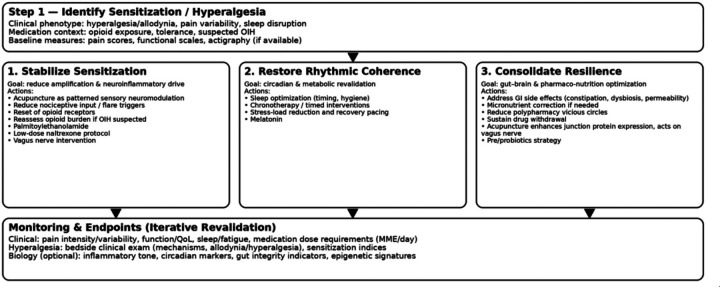
Therapeutic strategy for neuro-epigenetic revalidation in chronic pain. This figure presents a pragmatic multimodal strategy derived from the neuro-epigenetic revalidation framework. The approach is conceptualized as a layered process: (i) reduction of nociceptive amplification and neuroinflammatory drive (e.g., patterned sensory neuromodulation), (ii) restoration of circadian and metabolic coherence through chronobiological regulation, and (iii) consolidation of systemic resilience via gut–brain and pharmaco-nutritional optimization. Hyperalgesia is positioned as a measurable clinical marker of regulatory rigidity and as a potential therapeutic endpoint within this model.

Within this strategy, hyperalgesia becomes not only a symptom but a measurable therapeutic endpoint, assessable through clinical examination and biological markers proposed in this framework (Figure 2).

Methodologically, complex multimodal strategies require longitudinal and whole-system study designs capable of capturing temporal dynamics and interdependent mechanisms. Biomarkers findings should be interpreted cautiously, as associations do not equate to causality, and robust inference will require careful study design, repeated measures, and multimodal triangulation. Future research should focus on validating biomarker panels and integrating epigenetic, chronobiological, and gut–brain measures to support personalized, mechanism-based pain interventions (Figure 2).

By providing a coherent conceptual scaffold, the neuro-epigenetic revalidation model seeks to facilitate dialogue between clinical practice and translational research, without overstating current empirical evidence.

This integrative model is intended to generate testable hypothesis rather than to imply established causal mechanisms.

## 5 Conclusion

Chronic pain is increasingly recognized as a disorder of maladaptive system-level regulation sustained by interacting processes that include altered neural network connectivity, persistent neuroimmune activation, circadian disruption, metabolic dysregulation, and environmental mediated epigenetic modulation. These processes are not independent; rather, they form a self-reinforcing regulatory loop in which neuroinflammation promotes central sensitization, circadian misalignment amplifies inflammatory mediators and stress reactivity, metabolic strain impairs mitochondrial resilience, and epigenetic mechanisms stabilize these maladaptive states over time.

Within this context, non-pharmacological interventions should no longer be viewed solely as adjunctive or symptomatic therapies. Instead, they may be conceptualized as modulators of the regulatory nodes that sustain pain chronification. Patterned neuromodulator inputs such as acupuncture can engage descending inhibitory pathways and autonomic balance, potentially attenuating central sensitization and neuroimmune activation. Chronobiological regulation of sleep–wake cycles and light exposure may reduce stress-axis hyperactivation, normalize inflammatory oscillations, and restore temporal coherence across central and peripheral systems. Nutritional and pharmaco-metabolic strategies, including gut–brain modulation, may influence systemic inflammatory tone, intestinal barrier integrity, mitochondrial metabolism, and epigenetic regulation, thereby reduce biological vulnerability and enhance adaptive plasticity.

The neuro-epigenetic revalidation framework proposed here integrates these mechanisms within a unified systems model. Rather than targeting isolated molecular pathways, it emphasizes multimodal synergy: coordinated modulation across neural, immune, metabolic, and circadian domains that may progressively shift the organism from a rigid, sensitized state toward greater physiological flexibility. Epigenetic mechanisms provide a plausible biological substrate for this transition, as dynamic changes in chromatin accessibility and gene expression can link environmental and behavioural inputs to sustained regulatory recalibration.

Convergent improvements in inflammatory biomarkers, chronobiological stability, gut-related markers, epigenetic signatures, and multidimensional clinical outcomes would support the notion of systemic revalidation. Longitudinal, multimodal research designs are therefore essential to evaluate both mechanistic efficacy and real-world effectiveness.

By articulating the interconnected biological mechanisms linking neuroscience, epigenetics, chronobiology, and integrative medicine, the neuro-epigenetic revalidation paradigm offers more than an additive model of therapies. It proposes a mechanism-driven, personalized approach to chronic pain management aimed at restoring regulatory coherence and durable system-level resilience rather than merely suppressing nociceptive process.

## Data Availability

The data supporting the conclusions of this article will be made available by the authors, without undue reservation.
